# Assessment of Dietary Bioactive Phenolic Compounds and Agricultural Sustainability of an African Leafy Vegetable *Corchorus olitorius* L.

**DOI:** 10.3389/fnut.2021.667812

**Published:** 2021-07-01

**Authors:** Lorenzo Guzzetti, Davide Panzeri, Marynka Ulaszewska, Grazia Sacco, Matilde Forcella, Paola Fusi, Nicola Tommasi, Andrea Fiorini, Luca Campone, Massimo Labra

**Affiliations:** ^1^Department of Biotechnology and Bioscience, University of Milan-Bicocca, Milan, Italy; ^2^Department of Food Quality and Nutrition, Research and Innovation Center, Edmund Mach Foundation, Trento, Italy; ^3^Department of Sustainable Crop Production, Università Cattolica del Sacro Cuore, Piacenza, Italy

**Keywords:** indigenous vegetables, antioxidants, phenolic compounds, bioactivity, agronomic practices

## Abstract

*Corchorus olitorius* L. is an African leafy vegetable of high nutritional interest. To assess its agricultural suitability to sustainable cultivation conditions and its potential benefits for human nutrition, its phytochemical content in response to conservation agriculture practices [i.e., no-tillage (NT) and cover crop maintenance] and low water regime were evaluated and compared with response under conventional agriculture management. Hydric stress and NT did not affect the content of antioxidant metabolites, compared to conventional agricultural practices. In both conditions, leaves were found to be a great source of phenolic compounds. The effect of these phenolic fractions was assessed on two colon cell phenotypes to evaluate putative nutraceutical properties. Polyphenol-enriched extracts (PEEs) displayed selective cytotoxic activities against tumor Caco-2 cells but not on the healthy CCD841 line. PEEs were able to trigger oxidative stress and to inhibit the activity of glutathione-independent antioxidant enzymes on Caco-2 cells. *C. olitorius* showed to be a promising crop for improving both agricultural sustainability and health benefits due to the great amount of antioxidant compounds in leaves, whose occurrence is not altered by stressful farming conditions. Given its high adaptability, the cultivation of this crop is therefore recommendable also in the Mediterranean Basin.

## Introduction

The UN Sustainable Development Goals (i.e., SDG 2) highlight the need of identifying sustainable ways to produce food for human mankind as one of the major challenges of the Anthropocene era. As a matter of fact, hunger is still on the rise and many people consume low-quality food causing micronutrient deficiencies (1). Also in developed countries, the population is inadequately nourished and many environmental systems and processes are pushed beyond safe boundaries by food production. In this context, the scientific targets for healthy diets and sustainable food systems are integrated into a common framework of “one health”; therefore, the innovation of the agri-food system in the modern society allows to merge the issue of malnutrition and sustainability. Moreover, diet is not only aimed at guaranteeing a suitable source of macronutrients but also at ensuring a balanced intake of micronutrients (2), including bioactive compounds that can promote human health and solve global nutritional issues, such as hidden hunger, stunting and famine. Not only vitamins and minerals, but also dietary phytochemicals such as plant antioxidant, anti-inflammatory, and anti-aging compounds originating from leafy vegetables have been proved to contrast the outbreak of non-communicable diseases (NCDs), as well as metabolic disorders (e.g., obesity), so that their uptake is recommended by institutions such as the World Health Organization (WHO) (3–5). Leafy vegetables are sources of nutritional components, including proteins (6), dietary fibers (4), vitamins (7), and minerals (8), as well as natural antioxidants, including phytochemicals (9, 10).

In African rural communities, there is a wide range of indigenous vegetables cultivated by households for self-consumption that represent a valuable source of micronutrients and phytochemicals (2). The dietary diversification through the introduction of these food items in feeding programs is recommendable to reduce the risk of malnutrition and hidden hunger-related pathologies (6). However, the importance of identifying or resuming new species of agricultural interest does not deal only with nutritional and healthy needs, but also with environmental and sustainability concerns. This could be achieved by fostering the adoption of low-impact practices at the environmental level (11), such as conservation agriculture which is able to guarantee energetic, hydric and emissions savings (12, 13). In this work, we evaluated the healthy properties of *Corchorus olitorius* L., an indigenous African leafy vegetable known as jute mallow. This plant is largely diffused in the Middle East and in African countries (14–16) and is mainly cultivated in smallholding farms for self-consumption. It is made into a common mucilaginous soup or sauce in some West African cooking traditions (17). It has a high content of fiber, vitamins and antioxidant compounds (18), it is highly resistant to environmental stressors such as heat and water deficiency (2), and requires low fertilization to grow (8). We hypothesized that similarly to other leafy vegetables, stressful cultivation conditions, such as hydric stress, could trigger variations in the *C. olitorius* metabolic profile leading to higher polyphenolic and antioxidant compounds contents (19, 20). This may also have an impact on the nutraceutical properties of leaves.

The aims of the study are (i) to unravel the metabolic composition of jute mallow phenolic fractions obtained from leaves cultivated under different tillage and watering regimes in order to identify the most suitable and sustainable agronomic condition to enhance the leafy antioxidant content of this species, and (ii) to evaluate the bioactivity of the leafy phenolic fraction on human colon cell lines to unravel if the consumption of this leafy vegetable could be related to a reduced risk of gut malignancies, as already shown for other vegetables (21). This hypothesis is derived from a previous study (22), which highlighted a selective cytotoxic activity of jute mallow leaves against a liver cancer cell line (HepG2), with no effects on healthy FL83B cells. However, no information has been reported so far about the effect of such green leafy items on gut cancer and, more generally, on their potential role in enhancing human well-being.

## Materials and Methods

### Plant Material and Growing Conditions

Seeds of *C. olitorius* (accession ID. SUD-2) were obtained from the seed bank of the Word Vegetation Center (AVRDC, Tanzania). Plants were cultivated with two different agricultural approaches (conventional vs. conservation agriculture practices) following the same experimental design described in (13) through a randomized complete block design (RCBD) with two factors (i.e., tillage and irrigation with two levels each). Briefly, between May and August 2018, the trial took place in a long-term experimental field in San Bonico, Piacenza, Italy (45°00'18.05”N, 9°42'15.285”E; 68 m above sea level). Environmental temperatures ranged between 15 and 40°C and humidity was between 35 and 85%. A total of eight plots (5 × 3 m) were set up. Four of these were managed following the principles of conservation agriculture [i.e., 8 years of no-tillage (NT) and cover crops maintenance during the resting season of the field], while the other four were conventionally managed [i.e., conventional tillage (CT) and absence of cover crops]. Before sowing, all plots were fertilized at the same rate [50 kg nitrogen (N) ha^−1^], by applying urea (*N*-46%). The main physicochemical properties at the beginning of the experiment were pH 6.8, soil organic C 12.8 g kg^−1^, total N 1.2 g kg^−1^, available P 32 mg kg^−1^, exchangeable K 294 mg kg^−1^ and cation-exchange capacity 30 cmol kg^−1^. High phosphorus (P) and potassium (K) concentrations in the 0–30 cm soil layer [available P (Olsen): 32 mg kg^−1^; exchangeable K: 294 mg kg^−1^] suggested that P and K fertilization processes would not have been necessary according to the plant requirements (23). The soil is a fine, mixed, mesic Udertic Haplustalf (24) with a silty clay loamy texture (sand 122, silt 462, and clay 416 g kg^−1^) in the upper layer (0–30 cm), well-drained and non-saline. Plants were seeded at a distance of ~10–15 cm from each other with a total density of 40 individuals per plot. To test the ability of *C. olitorius* to resist against water stress, two separated subplots for each growing condition were defined (20 plants per subplot): one subplot was fully watered (FW), while the other was half-watered (HW) and protected from rain by applying greenhouses only during rainfalls (in order to induce stress without modifying the growth conditions of not-irrigated plants). Moreover, to avoid water percolation, the HW subplots were delimited by using steel gilders planted into the soil up to a depth of 30 cm. Specifically, the HW subplots received only 46% (113 mm) of the total water used for the irrigation of the FW subplots (245 mm). Stressful watering conditions for this species are joined with ~160–180 mm per growth season according to Maseko et al. (25). Just before flowering, leaves were manually collected and stored at−20°C before analyses.

### Phytoextraction

Pooled mixtures of leaves obtained for each subplot were processed in triplicate independently. In particular, leaves were washed and boiled in water at 100°C for 15 min in order to mimic African traditional culinary habits (17, 18, 26, 27). Leaves were dried in an oven at 50°C overnight and then grinded and stored at −20°C until analysis. Each pool of leaves was extracted according to the protocol of (28). Briefly, 1 g of powdered leaves was suspended in 4.8 mL of methanol 67% and 4.8 mL of chloroform. The powder was extracted on an orbital shaker (VDRL 711+, Asal, Italy) for 15 min at a constant rotation (400 rpm). Samples were extracted for a second time by adding a further 2.4 mL of the abovementioned extraction solution. The alcoholic portion of the extract was evaporated by using a rotary evaporator (40°C, 120 rpm, 1,000 bar); then, the remaining aqueous fraction was freeze-dried. Finally, samples were resuspended in water in order to obtain a concentration of extract equal to 6.7 mg/mL. All chemicals were purchased from Sigma-Aldrich Merck, Germany.

### Total Phenol Content Evaluation

The total phenol content of each extract was estimated by the Folin-Ciocalteau assay as described in (29) with minor modifications. Each sample was diluted to a concentration equal to 0.2 mg/mL and a calibration curve was made up by using increasing concentrations of gallic acid (from 0 to 100 μg/mL). The analysis was performed by adding in a quartz cell 400 μL of ultrapure milli-Q water, 80 μL of sample or standard, 40 μL of Folin-Ciocalteau reagent and 480 μL of 10.75% Na_2_CO_3_ solution. After an incubation of 30 min at room temperature, samples were read against the blank at a wavelength of 760 nm. Results are expressed as mg GAE (gallic acid equivalent) per gram of leaves. All chemicals were purchased from Sigma-Aldrich Merck, Germany.

### DPPH Radical Scavenging Activity

The radical scavenging activity of jute mallow total extracts was evaluated through the DPPH (2,2-diphenyl-1-picrylhydrazyl) assay as described in (29) with minor modifications. In brief, 50 μL of samples at a concentration of 0.2 mg/mL was added to 950 μL of 0.1 mM DPPH.

A calibration curve was made up by using Trolox (6-hydroxy-2,5,7,8-tetramethylchroman-2-carboxylic acid) as a standard reference in a range of concentrations between 0 and 500 μM. After an incubation of 30 min at room temperature, samples were read against the blank at a wavelength of 515 nm. Results are expressed as mg TE (Trolox Equivalent) per gram of leaves. All chemicals were purchased from Sigma-Aldrich Merck, Germany.

### Solid-Phase Extraction and UHPLC-DAD-MS Analysis

In order to enrich the polyphenolic fraction of jute mallow extracts, a SPE-based purification procedure was performed. Samples were loaded, washed (3 mL H_2_O), eluted (4.5 mL MeOH pH 2) and finally evaporated in a speed vacuum centrifuge (Eppendorf, Germany). Dried samples were resuspended (0.3 mg/mL) for UHPLC-DAD-MS analysis (ThermoFisher Scientific, Italy). Chromatographic separation was performed through a Kinetex Biphenyl column (50 × 2.1 mm, 2.6 μm, Phenomenex, USA) at a flow rate of 0.3 mL/min by gradient elution. Two wavelengths (280 and 330 nm) were employed for the detection and quantification of target analytes. ESI HRMS analysis, both in positive and in negative ion modes, was used for the characterization of the main phenolic compounds in the polyphenol-enriched extracts (PEEs). The ESI parameters and product ions scans were optimized to obtain a good response of polyphenols and their characteristic fragments. Detected compounds were tentatively identified as described in (30). A calibration external standard method was used to quantify the main phenolic compounds in PEEs. A mixture of eight standards—neochlorogenic acid (NCA, LOD: 1.178 μg/g; LOQ: 3.927 μg/g); cryptochlorogenic acid (CCA, LOD: 0.508 μg/g; LOQ: 1.738 μg/g); chlorogenic acid (CA; LOD: 0.354 μg/g; LOQ: 1.21 μg/g); quercetin-3-*O*-galactoside (Q3Gal, LOD: 2.12 μg/g; LOQ: 7.11 μg/g), isoquercitrin (Q3Gly, LOD: 1.2 μg/g; LOQ: 3.9 μg/g), quercetin-3-*O*-malonyl-glycoside (Q3MGly, LOD: 5.33 μg/g; LOQ: 17.73 μg/g), 3,5-dicaffeoylquinic acid (3,5-DCQA, LOD: 0.28 μg/g; LOQ: 0.964 μg/g), and kaempferol-3-O-glycoside (K3Gly, LOD: 4.87 μg/g; LOQ: 16.25 μg/g)—was used to produce a calibration curve (in a range between 1 and 100 μg/mL).

### Bioactivity Analysis

The eight most concentrated PEEs of the 16 plots were evaluated for their bioactive effects on healthy and colorectal cancer cell lines. The effect of each extract on the colon cells was evaluated by using CCD841 (ATCC^®^ CRL-1790™) human healthy intestinal mucosa cell line and Caco-2 (ATCC^®^ HTB-37™) human colorectal cancer cell line by MTT [3-(4,5-dimethylthiazol-2-yl)-2,5-diphenyltetrazolium bromide] assays and through oxidative stress analyses [reactive oxygen species (ROS) and antioxidant enzymes assessment]. Cells were grown in EMEM supplemented with heat-inactivated 10% fetal bovine serum (FBS), 2 mM _L_-glutamine, 1% nonessential amino acids, 100 U/mL penicillin, and 100 μg/mL streptomycin. All cell lines were maintained at 37°C in a humidified 5% CO_2_ incubator. ATCC cell lines were validated by short tandem repeat profiles that are generated by simultaneous amplification of multiple short tandem repeat loci and amelogenin (for gender identification). All the reagents for cell cultures were supplied by Lonza (Lonza Group, Basel, Switzerland).

#### Viability Assay

Cell viability assay was investigated using MTT-based *in vitro* toxicology assay kit (Sigma, St. Louis, MO, United States), according to the manufacturer's protocols.

The cell lines were seeded in 96-well microtiter plates at a density of 1 × 10^4^ cells/well, cultured in complete medium and treated after 24 h with 100 μg/mL of PEEs. After 48 h at 37°C, the medium was replaced with 100 μL of complete medium without phenol red containing 10 μL of 5 mg/mL MTT. After 4 h of further incubation for CCD841 and 2 h for CRC cell line, formazan crystals were solubilized with 10% Triton X-100 and 0.1 N HCl in isopropanol, and absorbance was measured at 570 nm using a microplate reader. Cell viability was expressed as a percentage against untreated cell lines used as controls.

#### Oxidative Stress Assay

The intracellular ROS and reactive nitrogen species (RNS) were detected by the oxidation of 2′,7′-dichlorofluorescin diacetate (H2DCFDA) (Sigma Chemical Co., St. Louis, MO). Caco-2 cell line was seeded in 96-well black microtiter plates at a density of 1 x 10^4^ cells/well, cultured in complete medium, and incubated after 24 h with 5 μM H2DCFDA in PBS for 30 min in the dark at 37°C. After two washes in PBS, cells were treated with 100 μg/mL of extract for 2 h or 1 mM H_2_O_2_ for 1 h for positive control. The fluorescence (λem = 485 nm/ λex = 535 nm) was measured at 37°C using a fluorescence microtiter plate reader (VICTOR X3, PerkinElmer).

#### Antioxidant Enzyme Activity Assessment

To evaluate the effect of PEEs on enzymatic activities, Caco-2 cell line was seeded at 1 × 10^6^ cells/100 mm dish and treated for 48 h with PEE at 100 μg/mL. Cells were rinsed with ice-cold PBS and lysed in 50 mM Tris–HCl pH 7.4, 150 mM NaCl, 5 mM EDTA, 10% glycerol, and 1% NP-40, containing protease inhibitors (1 μM leupeptin, 2 μg/mL aprotinin, 1 μg/mL pepstatin and 1 mM PMSF). Homogenates were obtained by passing five times through a blunt 20-gauge needle fitted to a syringe and then centrifuged at 15,000 × *g* for 30 min at 4°C. Supernatants were used to measure the enzymatic activities: Glutathione S-transferase (GST) was assayed as previously described by the method in the study by (31); glutathione reductase (GR) was assayed according to the method described in the study by (32); glutathione peroxidase (GPox) was assayed as reported in the study by (33); superoxide dismutase (SOD) was assayed as previously described in the study by (34); catalase (CAT) was assayed according to the method described in (35). All the experiments were normalized against an untreated control (CTRL). Enzymatic activities were expressed in international units and referred to protein concentration as determined by the Bradford method. All assays were performed in triplicate at 25°C in a Jasco V-550 Spectrophotometer.

### Statistical Analyses

All the data were analyzed using R version 3.3.3. Data from Folin-Ciocalteau and DPPH assays were analyzed by a Generalized Linear Mixed-Effect Model (GLMM) assuming a beta-binomial distribution of the response variable. Data from the MTT assay were analyzed again by a GLMM considering cell viability as a response variable and the cell line as an independent variable. The random effect was the plot which plants were grown in, and the fixed effect was the growth treatment. MTT data from single plots were analyzed by a Generalized Linear Model (GLM) with a quasi-binomial distribution of the response variable (% cell viability). The fixed effect was the cell line. To test the impact of the agronomic treatments on the metabolic profile of *C*. *olitorius*, data from HRMS were analyzed by comparing the intensity of the peaks both in positive and in negative mode through one-way ANOVA. Packages used were TMB, glmmTMB, and ggplot2. Data from enzymatic activity assays and ROS produced were analyzed by one-way ANOVA and Dunnett's *post-hoc* test through the multcomp package.

## Results

### Antioxidant Composition of *C. olitorius* Grown Under Different Agronomic Conditions

**Figure 1:**
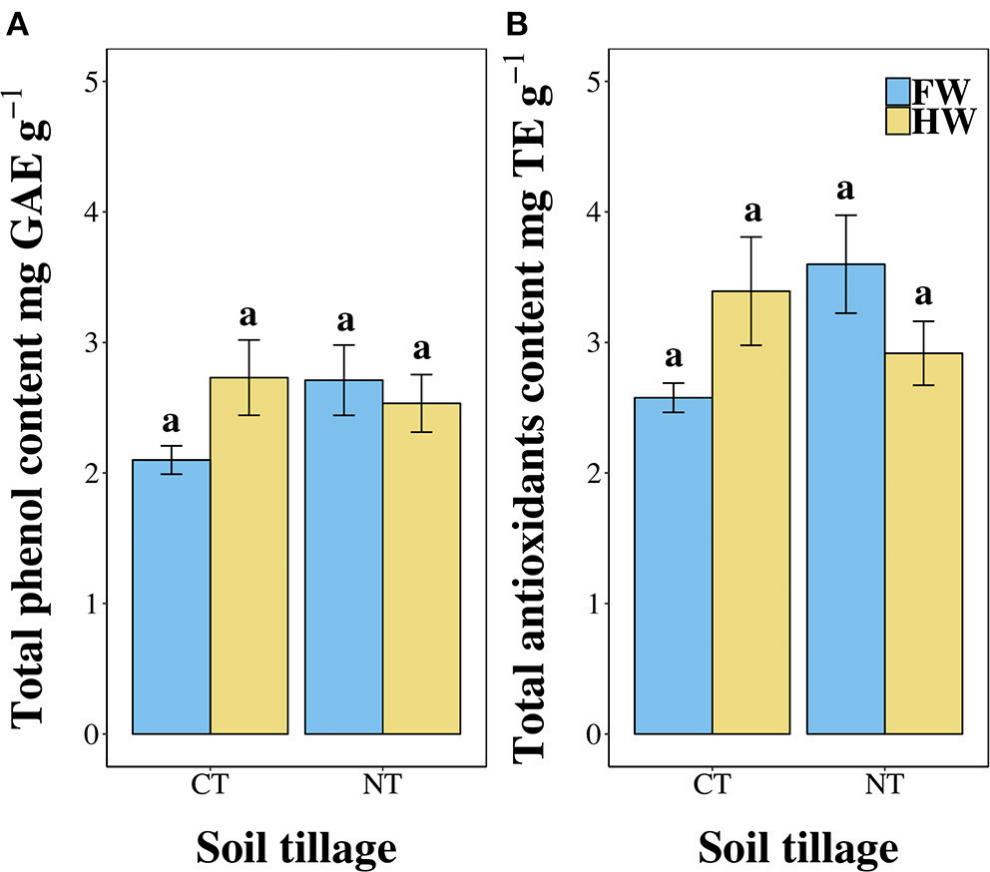
**(A)** Total phenol content (expressed as gallic acid equivalents per gram of dry leaves), **(B)** total antioxidants content (expressed as Trolox equivalents per gram of dry leaves) of jute mallow plants grown under the four different agronomic treatments. Data are expressed as the mean ± SEM. CT, conventionally tilled; NT, not-tilled; FW, fully watered; HW, half-watered.

Figures 1A,B show the total phenol content and total antioxidant composition of leaves harvested from plants grown under the different agronomic management. Data suggested that the total phenol content of boiled leaves of *C. olitorius* is around 0.25% of the total weight of leaves. No significant differences were detected among cultivation treatments (χ^2^ = 0.38, *p* = 0.944, Figure 1A). The radical scavenging activity followed the same trend identified for the total phenol content and, in particular, is comparable among treatments with values ranging between 2.5 and 3.2 mg TE per gram of leaves. Also in this case, none of the treatments was found to significantly affect the radical scavenging activity of the extracts (χ^2^ = 0.3, *p* = 0.9599; see Figure 1B).

### Qualitative and Quantitative Profiles of Jute Mallow Extract by UHPLC-DAD-HRMS^n^

In order to estimate the chemical composition of main compounds occurring in leafy extracts and to identify the compounds responsible for the abovementioned antioxidant activity (see Figure 1B), a HRMS untargeted screening was performed on the extract from each experimental growth condition. The leafy extracts were analyzed by UHPLC-DAD-HRMS^n^ to investigate their qualitative phenolic profile (Supplementary Table 1). Metabolite assignments were made by comparing retention times, UV/Vis spectra, and MS data (accurate mass and MS^n^ fragment ions) of the detected compounds with standards, whenever available, and jute mallow compounds reported in the literature and databases. The most abundant phenolic compounds are listed in Supplementary Table 1 with the main information (name, retention times, UV and MS data) for each component. The main compounds were quercetin and isomers of kaempferol-(malonyl-)-hexosides, chlorogenic acids, dicaffeoylquinic acids, and feruloyl-quinic acids. Results of HRMS untarget analysis did not reveal statistically significant differences between agricultural treatments (see Supplementary Table 1). Quantitative data are reported in Table 1, whereas Supplementary Figure 1 shows the phenolic profile of the main compounds both in standard solution and in PEEs at 280 (a) and 330 nm (b). A total of 11–20 peaks per sample were detected and eight of these peaks were identified. PEEs showed a total phenolic content calculated as the sum of individual phenolic compounds ranging between 74 and 99 μg/mL (with a range between 1,794.4 and 2,612.5 μg per gram of dry leaves). In particular, Q-3-O-MG was the most abundant phenolic compound in all the analyzed samples (32–39 μg/mL, with a range between 695.7 and 1,026.9 μg per gram of dry leaves). Conversely, the content of chlorogenic acid derivatives in all the treatments was very low, ranging between 0.18 and 0.65 μg/mL (4.32–53.7 μg per gram of dry leaves) as well as the level of K-3-O-Gly (1.9-3.3 μg/mL with a range between 34.3 and 86.2 μg per gram of dry leaves). In the case of these latter compounds, values display a high variability due to the fact that the presented data (Table 1) come from plants grown in different plots, although subjected to the same agronomic treatments. However, the results of the quantitative analysis did not show statistically significant differences among the investigated agronomic treatments.

**Table 1:** Concentration (μg/g) of the main phenolic species occurring within PEEs.

**Compounds (μg/g)**	**NTFW**	**CTFW**	**NTHW**	**CTHW**
NCA	7.6 ± 7.6	7.2 ± 7.2	<LOQ	4.9 ± 4.9
CCA	16.5 ± 10.3	11.8 ± 7.7	<LOQ	6.3 ± 6.3
CA	29.6 ± 15.5	13.9 ± 4.5	4.32 ± 4.32	15.0 ± 6.2
3,5-DCQA	77.5 ± 19.9	54.9 ± 32.4	50.4 ± 6.7	56.5 ± 0.7
Q-3-O-Gal	630.4 ± 69.7	525 ± 178.1	488.4 ± 40.3	768.2 ± 123.6
Q-3-O-Gly	574.8 ± 123.5	595.4 ± 43.5	398.1 ± 90.5	648.5 ± 69.4
Q-3-O-MG	819.6 ± 188.8	695.7 ± 188.4	805.5 ± 25.1	1,026.9 ± 205.0
K-3-O-Gly	67.9 ± 16.5	34.3 ± 29.3	47.7 ± 11.1	86.2 ± 6.0
Total	2,223.9	1,938.2	1,794.4	2,612.5

*Data represent the concentration of each compound occurring in 1 g dry leaves and are expressed as the mean ± SEM. For each cultivation condition, the quantification of the specific analytes was performed considering three biological replicates (i.e., leaves coming from plants cultivated in different plots). NTFW, not-tilled and fully watered plots; CTFW, conventionally tilled and fully watered plots; NTHW, not-tilled and half-watered plots; CTHW, conventionally tilled and half-watered plots. <LOQ: values not quantifiable since below the limit of quantification*.

### MTT Viability Assay

PEEs from the different experimental conditions were evaluated for their bioactive properties on CCD841, a healthy colon cell line used as a reference and Caco-2, a colorectal cancer cell line.

MTT assay performed on cells treated with PEEs showed significant effects (*p* < 0.001) on Caco-2 cells at a concentration of 100 μg/mL with an average viability reduction equal to 39%, compared with the healthy cell line, which maintained a percentage of viability higher than 80%; therefore, it was not negatively affected by the treatment with PEEs of *C. olitorius*.

**Figure 2:**
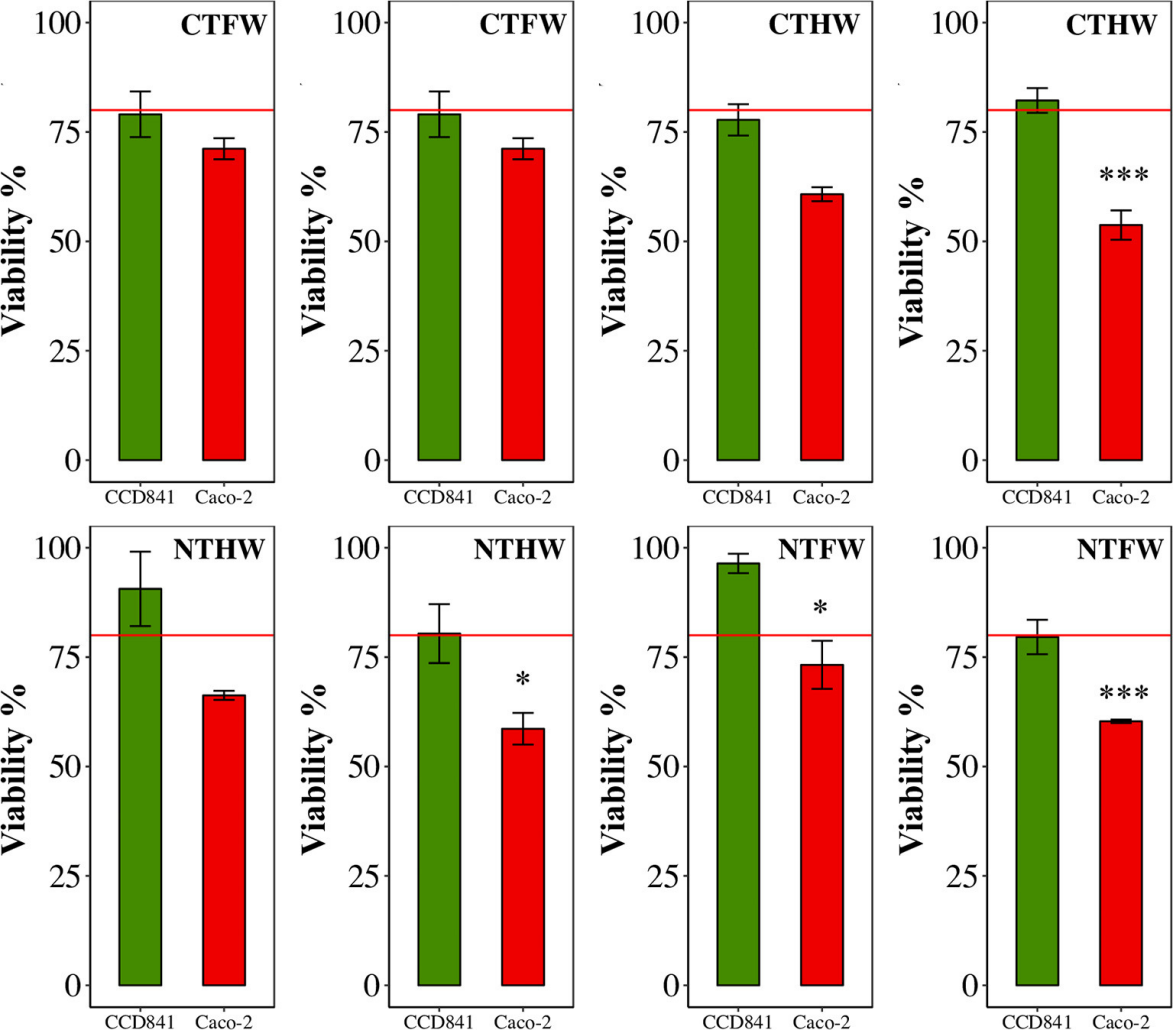
MTT viability assay on two human: cell lines: CCD841 (green, healthy line) and Caco-2 (red, colorectal cancer cell line). All cell lines were treated with 100 μg/mL GAE in the medium. Data are mean ± SEM. *p < 0.05, **p < 0.01, ***p < 0.001. CTFW, conventionally tilled and fully watered plots; CTHW, conventionally tilled and half-watered plots; NTHW, not-tilled and half-watered plots; NTFW, not-tilled and fully watered plots.

The analysis performed on single plots (Figure 2) highlighted a stronger effect on Caco-2 from plants grown under NT management. Three of the four plots analyzed showed a higher viability reduction in Caco-2 cell line than in healthy CCD841 cells. Only one plot from conventional agriculture management (CT) was found to be significantly more cytotoxic against Caco-2 cells compared with the healthy control. No significant effects were found to be driven by the drought treatment.

### Oxidative Stress Analysis

**Figure 3:**
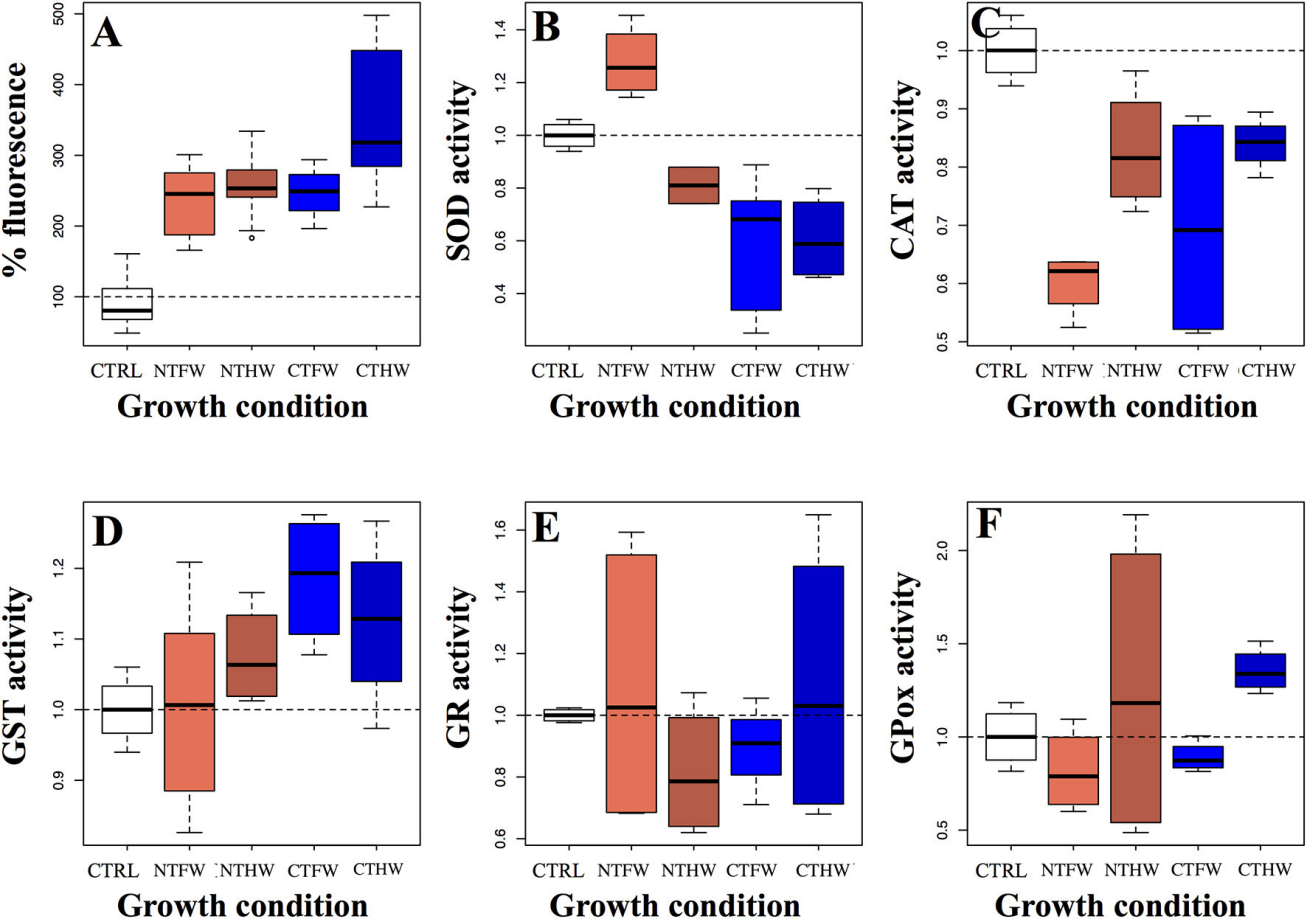
Oxidative stress detection **(A)** and enzymatic activity assays expressed as fold units compared to a non-treated control (CTRL) of five antioxidant cellular enzymes **(B)** superoxide dismutase, **(C)** catalase, **(D)** glutathione S-transferase, **(E)** glutathione reductase, **(F)** glutathione peroxidase performed on Caco-2 cells treated with 100 μg/mL GAE in the culture medium. CTFW, conventionally tilled and fully watered plots; CTHW, conventionally tilled and half-watered plots; NTHW, not-tilled and half-watered plots; NTFW, not-tilled and fully watered plots.

An oxidative stress analysis was performed to better clarify the action mechanism of jute mallow PEEs on cancer cells. The fluorescence analyses performed on Caco-2 cells revealed a significant increase in the production of ROS compared to the control (*F* = 38.53, *p* < 0.001) after 2-h treatment. Each tested sample induced the same effect with quantitative differences expressed in Figure 3A.

The intracellular detoxification systems were also evaluated to clarify the cellular response after PEE treatments in the Caco-2 cell line.

SOD activity was found to be significantly affected by PEE treatments (*F* = 11.54, *p* < 0.001; Figure 3B). In particular, SOD activity was negatively affected by the treatment with PEEs from tilled plots, both FW (*p* = 0.01) and HW (*p* = 0.023) compared to the control, while no significant effects were elicited by the treatment with PEEs from not-tilled plots, both FW (*p* = 0.562) and HW (*p* = 0.127).

CAT was found to be the most affected enzyme (*F* = 13.13, *p* < 0.001; Figure 3C). Almost all the conditions induced a significant reduction in its enzymatic activity, more marked from PEEs coming from the FW condition, both from CT (*p* < 0.001) and from NT (*p* < 0.001) treatment. PEEs from HW plots displayed a weaker effect, significant for those obtained from NT plots (*p* =0.04) and only marginally significant concerning those derived from CT conditions (*p* = 0.058).

GST activity was found to be slightly increased by PEE treatment (Figure 3D). However, the only experimental group showing a significant difference (*t* = 2.833, *p* = 0.037) compared with the untreated control was CTFW, which was the PEE group showing the least ability to discriminate with the healthy cell line (Figure 2). Conversely, the other groups showed a not significant difference in the GST activation compared to the control. Furthermore, despite some fluctuations among treatments, GR activity did not show any significant variation compared to the control in all treated samples (*F* = 0.67, *p* = 0.622; Figure 3E). Therefore, GR did not appear to be a candidate to explain the decrease in viability of Caco-2 cells treated with PEEs. Also, GPox activity was found not to be influenced by extracts obtained from plants deriving from the different growth treatments (*F* = 1.331, *p* = 0.304; Figure 3F). Overall, enzymes involved in glutathione metabolism did not display significant variations under PEE treatment compared to the control.

## Discussion

### Responses of *C. olitorius* Antioxidant Metabolites to Conservation Agriculture Management

According to FAO (36), conservation agriculture is one of the most promising management strategies for worldwide agroecosystems, since it allows to improve and sustain productivity, to increase profits and food security while preserving and enhancing environmental resources (37). In terms of agricultural sustainability in Mediterranean countries, NT is reported to enhance water soil content in response to climate change (13) and to preserve soil from erosion and leaching (38). In our work, we highlighted that *C. olitorius* is rich in several polyphenols and that their amount and composition are not modified by stressful cultivation conditions, such as NT and the reduction of water irrigation. Generally, environmental stressors, such as osmotic stress raised from water scarcity, may be able to elicit the production of secondary metabolites in leaves, as it happens in the case of vegetable amaranth (19), *A. tricolor* (20) and *C. olitorius* itself, although this species was found to greatly vary in its response depending on the genotype (14). A dedicated screening of these cultivars for their suitability to be grown in the Mediterranean context could also be performed to select the most promising accession in terms of the production of secondary metabolites.

The total antioxidant activity is associated with the concentration of plant endogenous antioxidants, such as glutathione, provitamin A, ascorbic acid, different types of pigments, and secondary compounds, including chlorophylls, carotenoids, xanthophylls, and polyphenols (30, 39). In accordance with the variation in the phenolic content of *C. olitorius* leaves, their total antioxidant activity was found not to significantly differ based on the cultivation treatments.

Concerning the nutritional properties of *C. olitorius* leaves, the main critical element is represented by the consumption habits that involve boiling the leaves. In the case of *C. olitorius*, recent data showed that the total polyphenols content of fresh leaves ranges from 5.41 to 7.78 mg GAE/g DW depending on the genotype (40). Considering these reference values, our data suggest that boiling reduced the amount of polyphenols by at least 55–65%. This is in accordance with a similar research conducted on other leafy vegetables (41). Currently, there are no indications of *C. olitorius* leaves consumption alternative to boiling. However, innovative technologies could be applied to identify the methods of preparation and consumption able to preserve antioxidant metabolites, such as microwave cooking processes that were found to be the suitable strategies to preserve (or also to ameliorate) the amount of phenolic compounds and the antioxidant capacity of several green leafy vegetables (9).

### Healthy Properties of *C. olitorius*

In this study, the phenolic fractions occurring in the leaves of *C. olitorius* were found to induce a significant reduction in the viability of Caco-2 cancer cells without any detrimental effect on the healthy cell line. These properties have been observed despite the preliminary boiling and make *C. olitorius* a promising candidate species in the field of nutritional prevention. A similar effect in terms of selectivity in the reduction of HepG2 liver cancer cell line viability was shown in a previous study performed by (22), which found that the mechanism of action promoted by jute mallow extracts is the mitochondria-dependent apoptosis pathway. In our study, the selective cytotoxic activity triggered by *C. olitorius* PEEs appeared to be mediated by a sudden increase in the ROS level, still high after 2-h treatment and through a successive decrease in the activity of glutathione-independent antioxidant enzymes. This activity may appear as a paradox because phenolic compounds are known to be among the best antioxidant phytochemicals. However, it is important to note that the tumor environment shows different redox status compared to healthy tissues and this study confirms previous observations highlighting that—despite their well-known antioxidant properties on healthy tissues—these secondary compounds can induce pro-oxidant responses on tumor cells, triggering programmed cell death mechanisms such as apoptosis (42, 43).

Actually, many flavonoids were identified within PEEs, mostly belonging to the family of quercetin derivatives (Table 1; Supplementary Figure 1). Similarly, other studies showed the occurrence of these metabolites in leafy vegetables, as documented, for instance, in drought-tolerant vegetable amaranth (22, 23) and also in *C. olitorius* leaves (20). As an example, Q-3-O-Gal accounted for 20–30% of the total phenolic content of PEEs. It is likely that this compound (together with the other quercetin derivatives) may be responsible for the selective cytotoxicity against the Caco-2 cell line with no detrimental effects on the healthy one. The anti-inflammatory and antioxidant properties of this compound extracted from jute mallow leaves have been already documented by (44).

Another noteworthy secondary metabolite identified in PEEs is the flavonoid astragalin (K-3-O-Gly), which was found in different amounts in approximately all PEEs. This compound has been reported to modulate inflammatory responses by regulating the expression of NF-κB, iNOS as well as cytokines and chemokines (COX-2, TNF-α, IL-10, and IL-6). Astragalin is also known to be an inhibitor of ERK-1/2 and Akt signaling; therefore, it is a significant compound against the proliferation of cancer cells (45).

Finally, CA and its isomers have also been identified in the majority of the analyzed PEEs and are probably involved in the selective cytotoxicity exerted by *C. olitorius* phenolic fractions, as previously shown by (46).

The last part of this study is focused on evaluating if cultivation conditions may impact on jute mallow cancer preventive abilities. Our results suggested that samples obtained from conservation agriculture plots are endowed with a higher selectivity (in terms of cytotoxicity) toward the cancer cell line compared to the healthy one. Considering that no significant differences in the polyphenolic composition among different field growing conditions were identified, we hypothesize that other unidentified compounds could play a synergic role in reducing the viability of cancer cells by acting on multiple cellular pathways. It is likely that other antioxidant compounds, displaying selective cytotoxic properties against cancer cell lines, have been elicited in response to the growth conditions (47).

The new trends in cancer prevention and therapy are directed at identifying combinations of different phytochemicals, acting simultaneously on several pathways to contrast cancer progression and to overcome drug resistance mechanisms typical of chemotherapy (48, 49). In this regard, deeper differential analyses will better clarify which compounds vary between the tested growth conditions leading to differential bioactive responses.

## Conclusion

In this study, we showed that *C. olitorius* is an African indigenous vegetable whose phenolic profile is not affected by stressful cultivation conditions such as conservation management and low hydric regime. *C. olitorius* leaves are a source of antioxidant compounds such as flavonoids and chlorogenic acids that can trigger a selective cytotoxicity against colorectal cancer cells, but not on healthy cells. Further investigations may be performed in order to unravel if other chemicals acting as antioxidants, such as carotenoids, vitamins and minerals, may be elicited by conservation management and hydric stress conditions. Overall, *C. olitorius* fully meets the interests of modern food sciences in terms of sustainability and health. Furthermore, this plant constitutes an important resource not only for developing countries, but also for the Mediterranean context, where its cultivation may be recommended in order to improve the nutritional status of both African and Western diets.

## Data Availability Statement

The original contributions presented in the study are included in the article/Supplementary Material, further inquiries can be directed to the corresponding author/s.

## Author Contributions

LG and ML designed the experiment. LG, DP, NT, and AF performed the field cultivation experiment. LG, LC, and MU performed the phytoextractions. LG, DP, LC, and MU chemically characterized the extracts. GS, MF, and PF performed the cellular assays. LG analyzed data. ML provided the financial support to the experiment. All authors significantly contributed to the writing of the manuscript.

## Conflict of Interest

The authors declare that the research was conducted in the absence of any commercial or financial relationships that could be construed as a potential conflict of interest.

## References

[1] Willett W Rockström J Loken B Springmann M Lang T Vermeulen S. Food in the anthropocene: the EAT–Lancet commission on healthy diets from sustainable food systems. Lancet. (2019) 393:447–92. 10.1016/S0140-6736(18)31788-4 30660336

[2] Padulosi S Thompson J Rudebjer P. Fighting Poverty, Hunger and Malnutrition with Neglected and Underutilized Species: Needs, Challenges and the Way Forward. Biodiversity International. (2013). Available online at: https://cgspace.cgiar.org/bitstream/handle/10568/68927/Fighting%20poverty%2C%20hunger%20and%20malnutrition%20with%20neglected%20and%20underutilized%20species%20%28NUS%29_1671.pdf?sequence=1&isAllowed=y (accessed November 15, 2020).

[3] Conti MV Campanaro A Coccetti P De Giuseppe R Galimberti A Labra. Potential role of neglected and underutilized plant species in improving women's empowerment and nutrition in areas of Sub-Saharan Africa. Nutr Rev. (2019) 77:817–28. 10.1093/nutrit/nuz038 31313806

[4] Sivakumar D Chen L Sultanbawa Y. A comprehensive review on beneficial dietary phytochemicals in common traditional southern african leafy vegetables. Food Sci Nutr. (2018) 6:714–27. 10.1002/fsn3.643 29983933 PMC6021739

[5] Maseko I Mabhaudhi T Tesfay S Araya HT Fezzehazion M Plooy CPD. African leafy vegetables: a review of status, production and utilization in South Africa. Sustainability. (2018) 10:1–16. 10.3390/su10010016

[6] Campanaro A Tommasi N Guzzetti L Galimberti A Bruni I Labra M. DNA barcoding to promote social awareness and identity of neglected, underutilized plant species having valuable nutritional properties. Food Res Int. (2019) 115:1–9. 10.1016/j.foodres.2018.07.031 30599919

[7] Sarker U Islam T Rabbani G Oba S. Genotype variability in composition of antioxidant vitamins and minerals in vegetable amaranth. Genetika. (2015) 47:85–96. 10.2298/GENSR1501085S

[8] Van Jaarsveld P Faber M Van Heerden I Wenhold F van Rensburg WJ Van Averbeke W. Nutrient content of eight african leafy vegetables and their potential contribution to dietary reference intakes. J Food Compos Anal. (2014) 33:77–84. 10.1016/j.jfca.2013.11.003

[9] Sergio L Boari F Pieralice M Linsalata V Cantore V Di Venere D. Bioactive phenolics and antioxidant capacity of some wild edible greens as affected by different cooking treatments. Foods. (2020) 9:1320. 10.3390/foods9091320 32962154 PMC7554971

[10] Sarker U Oba S. Nutraceuticals, antioxidant pigments, and phytochemicals in the leaves of *Amaranthus spinosus* and *Amaranthus viridis* weedy species. Sci Rep. (2019) 9:20413. 10.1038/s41598-019-50977-5 31892700 PMC6938518

[11] IPCC. Climate Change and Land: An IPCC Special Report on Climate Change, Desertification, Land Degradation, Sustainable Land Management, Food Security, and Greenhouse Gas Fluxes in Terrestrial Ecosystems. (2019). Available online at: https://www.ipcc.ch/srccl/ (accessed October 11, 2020).

[12] Dixit AK Agrawal RK Das SK Sahay CS Choudhary M Rai AK. Soil properties, crop productivity and energetics under different tillage practices in fodder +sorghum cowpea–wheat cropping system. Arch Agron Soil Sci. (2019) 65:492–506. 10.1080/03650340.2018.1507024

[13] Guzzetti L Fiorini A Panzeri D Tommasi N Grassi F Taskin. Sustainability Perspectives of *Vigna unguiculata* L. Walp. Cultivation under no tillage and water stress conditions. Plants. (2020) 9:48. 10.3390/plants9010048 31905903 PMC7020161

[14] Dhar P Ojha D Kar CS Mitra J. Differential response of tossa jute (*Corchorus olitorius*) submitted to water deficit stress. Ind Crops Prod. (2018) 112:141–50. 10.1016/j.indcrop.2017.10.044

[15] Kundu A Topdar N Sarkar D Sinha MK Ghosh A Banerjee. Origins of White (*Corchorus capsularis* L.) and Dark (C. *olitorius* L.) jute: a reevaluation based on nuclear and chloroplast microsatellites. J Plant Biochem Biotechnol. (2013) 22:372–81. 10.1007/s13562-012-0165-7

[16] Islam MM. Biochemistry, medicinal and food values of jute (*Corchorus capsularis* L. and *C. olitorius* L.) leaf: a review. Int J Enhanc Res Sci Technol Eng. (2013) 2:35–44. Available online at: www.erpublications.com

[17] Ola SS Catia G Marzia I Francesco VF Afolabi AA Nadia M. HPLC/DAD/MS characterization and analysis of flavonoids and cynnamoil derivatives in four nigerian green-leafy vegetables. Food Chem. (2009) 115:1568–74. 10.1016/j.foodchem.2009.02.013

[18] Zeghichi S Kallithraka S Simopoulos AP. nutritional composition of molokhia (*Corchorus olitorius*) and stamnagathi (*Cichorium spinosum*). World Rev Nutr Diet. (2003) 91:1–21. 10.1159/000069924 12747085

[19] Sarker U Oba S. Response of nutrients, minerals, antioxidant leaf pigments, vitamins, polyphenol, flavonoid and antioxidant activity in selected vegetable amaranth under four soil water content. Food Chem. (2018) 252:72–83. 10.1016/j.foodchem.2018.01.097 29478565

[20] Sarker U Oba S. Drought stress enhances nutritional and bioactive compounds, phenolic acids and antioxidant capacity of amaranthus leafy vegetable. BMC Plant Biology. (2018) 18:258. 10.1186/s12870-018-1484-1 30367616 PMC6203965

[21] Rajamanickam S Agarwal R. Natural products and colon cancer: current status and future prospects. Drug Dev Res. (2008) 69:460–71. 10.1002/ddr.20276 19884979 PMC2659299

[22] Li CJ Huang SY Wu MY Chen YC Tsang SF Chyuan H. Induction of apoptosis by ethanolic extract of *Corchorus olitorius* leaf in human hepatocellular carcinoma (HepG2) cells via a mitochondria-dependent pathway. Molecules. (2012) 17:9348–60. 10.3390/molecules17089348 22864242 PMC6268878

[23] Cooke GW. Fertilizing for Maximum Yield. 3rd ed. London; Sydney, NSW; Toronto, ON; New York, NY: Granada Publishing Ltd (1982).

[24] Soil Survey Staff. Keys to Soil Taxonomy. 12th ed. Washington, DC: Natural Resources Conservation Service of the United States Department of Agriculture (1982).

[25] Maseko I Ncube B Tesfay S Fessehazion M Modi AT Mabhaudhi T. Productivity of selected african leafy vegetables under varying water regimes. Agronomy. (2020) 10:916. 10.3390/agronomy10060916

[26] Moyo SM Serem JC Bester MJ Mavumengwana V Kayitesi E. African green leafy vegetables health benefits beyond nutrition. Food Rev Int. (2020) 37:1–18. 10.1080/87559129.2020.171751932912223

[27] Ngomuo M Stoilova T Feyissa T Ndakidemi PA. Characterization of morphological diversity of jute mallow (*Corchorus* spp.). Int J Agron. (2017) 2017:6460498. 10.1155/2017/6460498

[28] Vrhovsek U Masuero D Gasperotti M Franceschi P Caputi L Viola. A versatile targeted metabolomics method for the rapid quantification of multiple classes of phenolics in fruits and beverages. J. Agric Food Chem. (2012) 6:8831–8840. 10.1021/jf2051569 22468648

[29] Sarker U Hossain MM Oba S. Nutritional and antioxidant components and antioxidant capacity in green morph amaranthus leafy vegetable. Sci Rep. (2020) 10:1336. 10.1038/s41598-020-57687-3 31992722 PMC6987210

[30] Celano R Campone L Pagano I Carabetta S Di Sanzo R Rastrelli L. Characterisation of nutraceutical compounds from different parts of particular species of Citrus sinensis ‘Ovale Calabrese’by UHPLC-UV-ESI-HRMS. Nat Prod Res. (2019) 33:244–51. 10.1080/14786419.2018.1443102 29473425

[31] Habig WH Pabst MJ Jakoby WB. Glutathione S-Transferases the first enzymatic step in mercapturic acid formation. J Biol Chem. (1974) 249:7130–9. 10.1016/S0021-9258(19)42083-8 4436300

[32] Wang Y Oberley LW Murhammer DW. Antioxidant defense systems of two lipidopteran insect cell lines. Free Rad Biol Med. (2001) 30:1254–62. 10.1016/S0891-5849(01)00520-2 11368923

[33] Nakamura W Hosoda S Hayashi K. Purification and properties of rat liver glutathione peroxidase. Biochim Biophys Acta. (1974) 358:251–61. 10.1016/0005-2744(74)90455-028781

[34] Vance PG Keele BB Rajagopalan KV. Superoxide dismutase from streptococcus mutans isolation and characterization of two forms of the enzyme. J Biol Chem. (1972) 247:4782–86. 10.1016/S0021-9258(19)44979-X 4559499

[35] Jaworek D Gruber W Bergmeyer HU. Adenosine-5′-diphosphate and adenosine-5′-monophosphate. Methods Enzymat Anal. (1974) 2127–31. 10.1016/B978-0-12-091304-6.50066-5

[36] FAO. CA Adoption Worldwide. (2014). Available online at: http://www.fao.org/ag/ca/6c.html (accessed January 23, 2021).

[37] Boselli R Fiorini A Santelli S Ardenti F Capra F Maris C. Cover Crops during transition to no-till maintain yield and enhance soil fertility in intensive agro-ecosystems. Food Crop Res. (2020) 255:107871. 10.1016/j.fcr.2020.107871

[38] Dumanski J Peiretti R Benites JR McGarry D Pieri C. The paradigm of conservation agriculture. Proc World Assoc Soil Water Conserv. (2006) 1:58–64.

[39] Xu DP Li Y Meng X Zhou T Zhou Y Zheng J. J. Natural antioxidants in foods and medicinal plants: extraction, assessment and resources. Int J Mol Sci. (2017) 18:96. 10.3390/ijms18010096 PMC5297730 28067795

[40] Biswas A Dey S Li D Yiu L Zhang J Huang. Comparison of phytochemical profile, mineral content, and in vitro antioxidant activities of corchorus capsularis and corchorus olitorius leaf extracts from different populations. J Food Qual. (2020) 2020:2931097. 10.1155/2020/2931097

[41] Gunathilake KD Ranaweera KKD Rupasinghe HP. Effect of different cooking methods on polyphenols, carotenoids and antioxidant activities of selected edible leaves. Antioxidants. (2018) 7:117. 10.3390/antiox7090117 30200223 PMC6162770

[42] Lambert JD Elias RJ. The antioxidant and pro-oxidant activities of green tea polyphenols: a role in cancer prevention. Arch Biochem Biophys. (2010)501:65–72. 10.1016/j.abb.2010.06.013 20558130 PMC2946098

[43] León-González AJ Auger C Schini-Kerth VB. Pro-Oxidant activity of polyphenols and its implication on cancer chemoprevention and chemotherapy. Biochem Pharmacol. (2015) 98:371–80. 10.1016/j.bcp.2015.07.017 26206193

[44] Handoussa H Hanafi R Eddiasty I El-Gendy M El Khatib A Linscheid. Anti-inflammatory and cytotoxic activities of dietary phenolics isolated from *Corchorus olitorius* and *Vitis vinifera*. J Funct Foods. (2013) 5:1204–16. 10.1016/j.jff.2013.04.003

[45] Riaz A Rasul A Hussain G Zahoor MK Jabeen F Subhani. Astragalin: a bioactive phytochemical with potential therapeutic activities. Adv Pharmacol Sci. (2018) 2018:9794625. 10.1155/2018/9794625 29853868 PMC5954929

[46] Sadeghi Ekbatan S Li XQ Ghorbani M Azadi B Kubow S. Chlorogenic acid and its microbial metabolites exert anti-proliferative effects, s-phase cell-cycle arrest and apoptosis in human colon cancer caco-2 cells. Int J Mol Sci. (2018) 19:723. 10.3390/ijms19030723 29510500 PMC5877584

[47] Lee WL Huang JY Shyur LF. Phytoagents for cancer management: regulation of nucleic acid oxidation, ros, related mechanisms. Oxid Med Cell Longev. (2013) 2013:925804. 10.1155/2013/925804 24454991 PMC3886269

[48] Panzeri D Guzzetti L Sacco G Tedeschi G Nonnis S Airoldi. Effectiveness of *Vigna unguiculata* seed extracts in preventing colorectal cancer. Food Funct. (2020) 11:5853–5865. 10.1039/D0FO00913J 32589172

[49] Bukowski K Kciuk M Kontek R. Mechanisms of multidrug resistance in cancer chemotherapy. Int J Mol Sci. (2020) 21:3233. 10.3390/ijms21093233 32370233 PMC7247559

