# Supplementary material for: Assessment of Dietary Bioactive Phenolic Compounds and Agricultural Sustainability of an African Leafy Vegetable Corchorus olitorius L
Source: Front Nutr. 2021 Jul 1;8:667812. doi: 10.3389/fnut.2021.667812 (PMC8280468; doi:10.3389/fnut.2021.667812)
Supplement: Supplementary file 1 [file Data_Sheet_1.docx]

| **Compound** | **Rt (min)** | **Elemental Formula** | **Ion** | **m/z (Relative Intensity) ^a^** | **Annotation** | **MS/MS^2^ and MS/MS^3^ spectra** | **Significance** |
| --- | --- | --- | --- | --- | --- | --- | --- |
| Quercetin – malonyl-hexose isomers | 5.51  5.89  6.35 | C_24_H_22_O_15_ | + | 551.1031 (100)  552.1071 (30)  553.1093 (9)  303.0490 (25) | [M+H]^+^  [M+H]-C_9_H_12_O_8_]^+^ | MS^2^ 551: 303.0490 (100)  MS^3^ 551-303: 285.0387 (50); 257.0437 (100); 247.0594 (25); 229.0490 (100); 165.0178 (80); 137.0230 (22) | *F* = 1.151, *p* = 0.368 |
|  |  |  | - | 549.0879 (100)  550.0910 (30)  551.0932 (9) | [M-H]^-^ |  | *F* = 0.129, *p* = 0.941 |
| Chlorogenic acids isomers | 3.12  3.71  3.85  4.24 | C_16_H_18_O_9_ | + | 355.1027 (100)  356.1064 (19)  357.1082 (4)  163.0387 (30)  164.0421 (3) | [M+H]^+^ | @Rt 3.12 MS^2^ 355: 163.0383 (100); 145.0278(15)  @Rt 3.71 MS^2^ 355: 163.0383 (100); 145.0278(8)  @Rt 3.85 MS^2^ 355: 163.0383 (100); 145.0278(15) | *F* = 0.136, *p* = 0.937 |
|  |  |  | - | 353.0870 (100)  354.0901(20)  355.0921 (4)  191.0557 (35)  192.0591(3)  179.0343 (30)  180.0380 (3) | [M-H]^-^ | @Rt 3.12 MS^2^ 353: 191.0556(100); 179.0347 (60); 135.0449 (29); 173.0451 (10)  @Rt 3.71 MS^2^ 353: 191.0556(100); 179.0347 (10);  @Rt 3.85 MS^2^ 353: 173.0451 (100); 191.0556(80); 179.0347 (65); 135.0449 (29); 135.0447 (10)  @Rt 4.24 MS^2^ 353: 191.0556(100); 179.0347 (8); 135.0449 (5); | *F* = 0.425, *p* = 0.739 |
| Quercetin – glycoside  isomers | 4.99  5.10  5.23 | C_21_H_20_O_12_ | + | 465.1033 (100)  466.1067 (24)  467.1089 (5)  303.0495 (20) | [M+H]^+^ | MS^2^ 465: 303.0490 (100)  MS^3^ 465-303: 285.0386 (55); 257.0436 (100); 247.0594 (30); 229.0490 (100); 165.0179 (60); 137.0230 (20) | *F* = 1.164, *p* = 0.364 |
| Tetrahydroxyisoflavone malonyl-hexose | 6.55  6.64  6.79  7.18 | C_24_H_22_O_14_ | + | 535.1080 (100)  536.1120 (30)  537.1144 (6)  287.0545 (30) | [M+H]^+^  [M+H]-C_9_H_12_O_8_]^+^ | MS^2^ 535: 287.0545 (100)  MS^3^ 535-287: 269.0437 (20); 258.0516 (30); 241.0488 (90); 231.0647(30); 213.0540(100); 177.0670(50); 165.01777(100); 153.0179(60); 133.0281(20); 121.0280(40); 111.0077(15) | *F* = 1.151, *p* = 0.368 |
|  |  |  | - | 533.0931 (100)  534.0965 (30)  535.0985 (6) | [M-H]^-^ | MS^2^ 533: 285.0408 (100) | *F* = 0.386, *p* = 0.765 |
| Dicaffeoylquinic acid  isomers | 5.60,  5.90,  6.20,  6.50,  7.41 | C_25_H_24_O_12_ | + | 517.1329 (100)  5181362 (30)  519.1387 (6) | [M+H]^+^ | MS^2^ 517: 499.1217 (100)  MS^3^ 535-499:319.0803 (100); 163.0384(100); 145.0279(10) | *F* = 0.966, *p* = 0.44 |
|  |  |  | - | 515.1181 (100)  516.1213 (30)  517.1237 (6) | [M-H]^-^ | MS^2^ 515: 353.0864(100); 299.0549 (10); 203.0344(10); 173.0449 (15); 191.0533(15); 179.03410 (10); 255.0657 (15) | *F* = 1.364, *p* = 0.301 |
| Kaempferol hexoside isomers | 5.32,  5.70,  6.07 | C_21_H_20_O_11_ | + | 449.1080 (100)  450.1115 (25)  451.1140 (5) | [M+H]^+^ | MS^2^ 449: 287.0544 (100)  MS^3^ 449-287: 258.0512 (20); 241.0488 (80); 213.0539(80); 177.0669(75); 165.01777(100); 153.0178(63); 133.0280(20); 121.0280(40); 111.0077(15) | *F* = 0.835, *p* = 0.5 |
|  |  |  | - | 447.0922 (100)  448.0959 (25)  449.0983 (5) | [M-H]^-^ | MS^2^ 447: 327.0499 (20); 284.0315(100); 285.0393(60); 255.0290(13); 227.0341(15); 151.0031(10)  MS^3^ 449-284: 255.0293 (100) | *F* = 0.687, *p* = 0.577 |
| Feruoylquinic acid | 3.97,  4.46,  5.10 | C_17_H_20_O_9_ | + | 367.1027 (100)  368.1060 (20)  369.1079 (4) | [M+H]^-^ | MS^2^ 367: 191.0556 (100); 173.0451(45) | *F* = 0.565, *p* = 0.649 |

^a^ m/z italics are isotopes

n.a – not available

**Table S1** Results of HRMS untargeted screening: selection of peaks characterized by high intensity in positive and negative ionization modes. Comparison of MS/MS spectra performed with mzCloud database. Significance is referred to ANOVA analysis performed to compare agronomic treatments with a *p* value threshold set at 0.05.

Std. mix 25 mg/L

NTFW

NTHW

CTFW

CTHW

A

B

8

7

6

4

5

3

2

1

8

7

6

5

4

3

2

1

1

**Supplementary Figure 1** Representative chromatograms of standards solution and samples at (a) 280 nm and (b) 330 nm. (1): NCA, (2): CCA; (3) CA; (4): 3,5-DCQA; (5): Q-3-O-Gal; (6): Q-3-O-Gly; (7): Q-3-O-MG; (8): K-3-O-Gly.
